# 2,2-Dichloro-1-(3,3,6-trimethyl-9-oxo-1,5-diaza­bicyclo­[4.3.0]nonan-5-yl)ethanone

**DOI:** 10.1107/S1600536811027176

**Published:** 2011-07-13

**Authors:** Ying Fu, Fei Ye

**Affiliations:** a College of Science, Northeast Agricultural University, Harbin 150030, People’s Republic of China

## Abstract

In the title mol­ecule, C_12_H_18_Cl_2_N_2_O_2_, the six-membered ring is in a chair conformation and the five-membered ring is in an envelope conformation. In the crystal, weak inter­molecular bifurcated (C—H)_2_⋯O hydrogen bonds connect mol­ecules into chains along [010].

## Related literature

For synthetic applications of 1,5-diaza­bicyclo compounds, see: Hutton & Bartlett (2007) ▶; Koptelov *et al.* (2011 ▶); Taylor *et al.* (2010 ▶). For the bioactivity of *N*-dichloro­acety diaza­bicyclo derivatives, see: Burton *et al.* (1994 ▶); Hatzios (2004 ▶); Loniovereror (1993 ▶). For the synthetic procedure, see: Sun & Ye (2010 ▶).
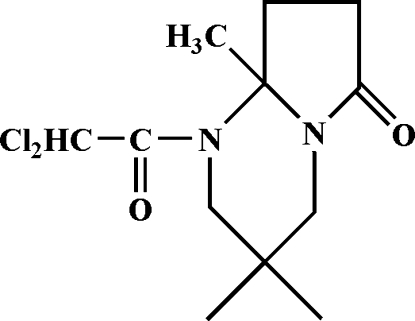


## Experimental

### 

#### Crystal data

C_12_H_18_Cl_2_N_2_O_2_
*M* *_r_* = 293.18
Monoclinic, 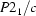

*a* = 9.4442 (18) Å
*b* = 14.116 (3) Å
*c* = 11.7555 (16) Å
β = 115.067 (11)°
*V* = 1419.6 (4) Å^3^
*Z* = 4
Mo *K*α radiation
μ = 0.45 mm^−1^
*T* = 298 K
0.42 × 0.40 × 0.28 mm

#### Data collection

Bruker SMART CCD diffractometer
Absorption correction: multi-scan (*SADABS*; Sheldrick, 1996 ▶) *T*_min_ = 0.832, *T*_max_ = 0.884
10829 measured reflections
3492 independent reflections
2556 reflections with *I* > 2σ(*I*)
*R*_int_ = 0.027

#### Refinement

*R*[*F* ^2^ > 2σ(*F* ^2^)] = 0.044
*wR*(*F* ^2^) = 0.129
*S* = 1.04
3492 reflections
166 parameters
H-atom parameters constrained
Δρ_max_ = 0.33 e Å^−3^
Δρ_min_ = −0.48 e Å^−3^

### 

Data collection: *SMART* (Bruker, 1998 ▶); cell refinement: *SAINT* (Bruker, 1998 ▶); data reduction: *SAINT*; program(s) used to solve structure: *SHELXS97* (Sheldrick, 2008 ▶); program(s) used to refine structure: *SHELXL97* (Sheldrick, 2008 ▶); molecular graphics: *SHELXTL* (Sheldrick, 2008) ▶; software used to prepare material for publication: *SHELXTL*.

## Supplementary Material

Crystal structure: contains datablock(s) global, I. DOI: 10.1107/S1600536811027176/lh5271sup1.cif

Structure factors: contains datablock(s) I. DOI: 10.1107/S1600536811027176/lh5271Isup2.hkl

Supplementary material file. DOI: 10.1107/S1600536811027176/lh5271Isup3.cml

Additional supplementary materials:  crystallographic information; 3D view; checkCIF report

## Figures and Tables

**Table 1:** Hydrogen-bond geometry (Å, °)

*D*—H⋯*A*	*D*—H	H⋯*A*	*D*⋯*A*	*D*—H⋯*A*
C1—H1⋯O2^i^	0.98	2.23	3.200 (3)	170
C3—H3*B*⋯O2^i^	0.97	2.50	3.390 (2)	153

Symmetry code: (i) 
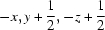
.

**Fig. 1.**
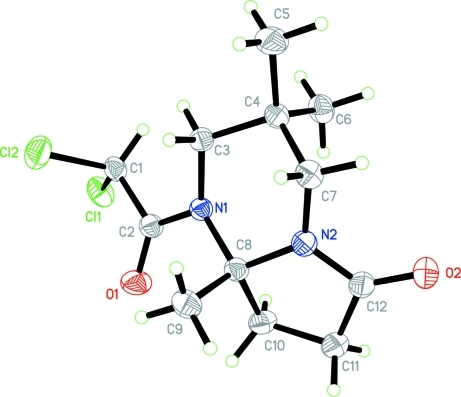
The molecular structure of the title compound with displacement ellipsoids drawn at the 30% probability level.

**Fig. 2.**
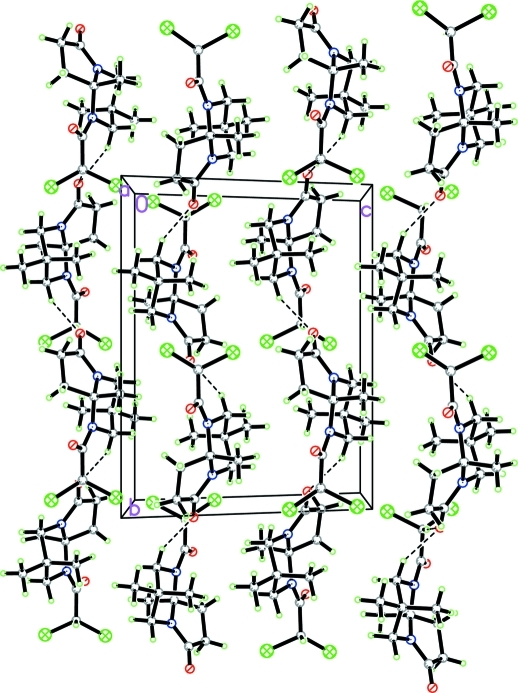
Part of the crystal structure showing the weak intermolecular C—H···O hydrogen bonds as dashed lines.

## supplementary crystallographic information

### Comment

1,5-Diazabicyclo compounds are important synthetic targets due to their biological activity (Hutton & Bartlett, 2007, Koptelov *et al.*, 2011) and catalytic activity (Taylor *et al.*, 2010). It was discovered that *N*-dichloroacetyl-1,5-diazabicyclo compounds act as herbicide safeners and these compounds have drawn widespread attention in agricultural biochemistry (Burton *et al.*, 1994, Hatzios, 2004, Loniovereror, 1993). As a part of our ongoing investigation on the bioactivities of safeners we have determined the crystal structure of the title compound.

The molecular structure of the title compound is shown in Fig. 1. In the crystal, weak interolecular bifurcated (C—H)_2_···O hydrogen bonds connect molecules into one-dimensional chains along [010]. (Fig. 2).

### Experimental

The title compound was prepared according to the literature procedure (Sun *et al.*, 2010). The single-crystal suitable for X-ray structural analysis was obtained by slow evaporation of a solution of the title compound in petroleum ether and ethyl acetate at room temperature.

### Refinement

All H atoms were initially located in a difference Fourier map. The C—H atoms were then constrained to an ideal geometry, with C—H distances of 0.96-98 Å, and *U*_iso_(H) = 1.2-1.5*U*_eq_(C). There is a relatively short H···H contact ca. 1.87Å. This appears to be influenced by the hydrogen bonding.

### Crystal data

**Table:**

C_12_H_18_Cl_2_N_2_O_2_	*F*(000) = 616
*M**_r_* = 293.18	*D*_x_ = 1.372 Mg m^−^^3^
Monoclinic, *P*2_1_/*c*	Mo *K*α radiation, λ = 0.71073 Å
Hall symbol: -P 2ybc	Cell parameters from 3515 reflections
*a* = 9.4442 (18) Å	θ = 2.4–27.1°
*b* = 14.116 (3) Å	µ = 0.45 mm^−^^1^
*c* = 11.7555 (16) Å	*T* = 298 K
β = 115.067 (11)°	Block, colourless
*V* = 1419.6 (4) Å^3^	0.42 × 0.40 × 0.28 mm
*Z* = 4	

### Data collection

**Table:**

Bruker SMART CCD diffractometer	3492 independent reflections
Radiation source: fine-focus sealed tube	2556 reflections with *I* > 2σ(*I*)
graphite	*R*_int_ = 0.027
φ and ω scans	θ_max_ = 28.3°, θ_min_ = 2.4°
Absorption correction: multi-scan (*SADABS*; Sheldrick, 1996)	*h* = −12→12
*T*_min_ = 0.832, *T*_max_ = 0.884	*k* = −18→18
10829 measured reflections	*l* = −9→15

### Refinement

**Table:**

Refinement on *F*^2^	Primary atom site location: structure-invariant direct methods
Least-squares matrix: full	Secondary atom site location: difference Fourier map
*R*[*F*^2^ > 2σ(*F*^2^)] = 0.044	Hydrogen site location: inferred from neighbouring sites
*wR*(*F*^2^) = 0.129	H-atom parameters constrained
*S* = 1.04	*w* = 1/[σ^2^(*F*_o_^2^) + (0.0683*P*)^2^ + 0.2384*P*] where *P* = (*F*_o_^2^ + 2*F*_c_^2^)/3
3492 reflections	(Δ/σ)_max_ < 0.001
166 parameters	Δρ_max_ = 0.33 e Å^−^^3^
0 restraints	Δρ_min_ = −0.48 e Å^−^^3^

### Special details

**Table:**

Geometry. All e.s.d.'s (except the e.s.d. in the dihedral angle between two l.s. planes) are estimated using the full covariance matrix. The cell e.s.d.'s are taken into account individually in the estimation of e.s.d.'s in distances, angles and torsion angles; correlations between e.s.d.'s in cell parameters are only used when they are defined by crystal symmetry. An approximate (isotropic) treatment of cell e.s.d.'s is used for estimating e.s.d.'s involving l.s. planes.
Refinement. Refinement of *F*^2^ against ALL reflections. The weighted *R*-factor *wR* and goodness of fit *S* are based on *F*^2^, conventional *R*-factors *R* are based on *F*, with *F* set to zero for negative *F*^2^. The threshold expression of *F*^2^ > σ(*F*^2^) is used only for calculating *R*-factors(gt) *etc*. and is not relevant to the choice of reflections for refinement. *R*-factors based on *F*^2^ are statistically about twice as large as those based on *F*, and *R*- factors based on ALL data will be even larger.

### Fractional atomic coordinates and isotropic or equivalent isotropic displacement parameters (Å^2^)

**Table:**

	*x*	*y*	*z*	*U*_iso_*/*U*_eq_	
Cl1	0.38732 (6)	−0.01048 (4)	0.34216 (5)	0.05901 (18)	
Cl2	0.21200 (7)	−0.01735 (4)	0.07141 (6)	0.0696 (2)	
O1	0.43042 (15)	−0.18995 (10)	0.24212 (16)	0.0618 (4)	
O2	0.06902 (18)	−0.54370 (10)	0.24058 (16)	0.0638 (4)	
N1	0.17907 (15)	−0.24099 (9)	0.17670 (13)	0.0342 (3)	
N2	0.10521 (16)	−0.40443 (10)	0.15860 (14)	0.0388 (3)	
C1	0.2408 (2)	−0.07096 (12)	0.21585 (17)	0.0419 (4)	
H1	0.1428	−0.0703	0.2257	0.050*	
C2	0.29300 (19)	−0.17366 (12)	0.21273 (17)	0.0399 (4)	
C3	0.01328 (18)	−0.21728 (12)	0.09737 (15)	0.0364 (4)	
H3A	−0.0029	−0.2147	0.0102	0.044*	
H3B	−0.0089	−0.1549	0.1205	0.044*	
C4	−0.10098 (18)	−0.28847 (12)	0.10984 (15)	0.0366 (4)	
C5	−0.2656 (2)	−0.26170 (16)	0.0151 (2)	0.0581 (5)	
H5A	−0.2699	−0.2615	−0.0679	0.087*	
H5B	−0.2913	−0.1998	0.0346	0.087*	
H5C	−0.3392	−0.3070	0.0193	0.087*	
C6	−0.0909 (2)	−0.28828 (14)	0.24271 (18)	0.0485 (4)	
H6A	−0.1645	−0.3329	0.2480	0.073*	
H6B	−0.1148	−0.2261	0.2627	0.073*	
H6C	0.0128	−0.3057	0.3011	0.073*	
C7	−0.0579 (2)	−0.38544 (12)	0.07744 (17)	0.0419 (4)	
H7A	−0.1235	−0.4337	0.0893	0.050*	
H7B	−0.0742	−0.3866	−0.0098	0.050*	
C8	0.22976 (19)	−0.33990 (11)	0.16558 (16)	0.0367 (4)	
C9	0.2611 (2)	−0.34929 (15)	0.04896 (19)	0.0516 (5)	
H9A	0.2991	−0.4119	0.0457	0.077*	
H9B	0.3380	−0.3035	0.0525	0.077*	
H9C	0.1660	−0.3384	−0.0247	0.077*	
C10	0.3679 (2)	−0.37342 (13)	0.28688 (18)	0.0477 (4)	
H10A	0.4655	−0.3695	0.2784	0.057*	
H10B	0.3763	−0.3353	0.3582	0.057*	
C11	0.3290 (2)	−0.47623 (14)	0.3029 (2)	0.0531 (5)	
H11A	0.3784	−0.5195	0.2666	0.064*	
H11B	0.3628	−0.4917	0.3909	0.064*	
C12	0.1545 (2)	−0.48091 (12)	0.23385 (19)	0.0454 (4)	

### Atomic displacement parameters (Å^2^)

**Table:**

	*U*^11^	*U*^22^	*U*^33^	*U*^12^	*U*^13^	*U*^23^
Cl1	0.0585 (3)	0.0609 (3)	0.0632 (3)	−0.0227 (2)	0.0313 (3)	−0.0199 (2)
Cl2	0.0747 (4)	0.0676 (4)	0.0629 (4)	0.0028 (3)	0.0257 (3)	0.0219 (3)
O1	0.0345 (7)	0.0509 (8)	0.0934 (12)	−0.0021 (6)	0.0206 (7)	−0.0014 (7)
O2	0.0683 (9)	0.0460 (7)	0.0921 (12)	0.0003 (7)	0.0483 (9)	0.0128 (8)
N1	0.0325 (7)	0.0336 (6)	0.0362 (7)	−0.0005 (5)	0.0141 (6)	−0.0005 (5)
N2	0.0405 (8)	0.0346 (7)	0.0426 (8)	−0.0015 (6)	0.0189 (6)	−0.0034 (6)
C1	0.0399 (9)	0.0389 (9)	0.0505 (10)	−0.0074 (7)	0.0226 (8)	−0.0032 (8)
C2	0.0360 (9)	0.0392 (8)	0.0431 (9)	−0.0025 (7)	0.0156 (8)	−0.0012 (7)
C3	0.0345 (8)	0.0391 (8)	0.0329 (8)	0.0000 (6)	0.0116 (7)	0.0019 (7)
C4	0.0336 (8)	0.0387 (8)	0.0359 (8)	−0.0028 (6)	0.0133 (7)	−0.0029 (7)
C5	0.0363 (10)	0.0621 (12)	0.0633 (13)	−0.0026 (9)	0.0089 (9)	0.0008 (10)
C6	0.0567 (11)	0.0495 (10)	0.0487 (11)	−0.0041 (8)	0.0313 (9)	−0.0047 (8)
C7	0.0418 (9)	0.0404 (9)	0.0412 (9)	−0.0073 (7)	0.0153 (8)	−0.0097 (7)
C8	0.0367 (8)	0.0344 (8)	0.0414 (9)	−0.0001 (6)	0.0189 (7)	−0.0026 (7)
C9	0.0573 (11)	0.0563 (11)	0.0524 (11)	0.0039 (9)	0.0342 (10)	−0.0030 (9)
C10	0.0419 (10)	0.0461 (10)	0.0507 (11)	0.0072 (8)	0.0153 (9)	0.0024 (8)
C11	0.0530 (11)	0.0509 (11)	0.0594 (12)	0.0123 (9)	0.0278 (10)	0.0129 (9)
C12	0.0565 (11)	0.0381 (9)	0.0533 (11)	0.0051 (8)	0.0345 (10)	0.0008 (8)

### Geometric parameters (Å, °)

**Table:**

Cl1—C1	1.7627 (18)	C5—H5B	0.9600
Cl2—C1	1.7715 (19)	C5—H5C	0.9600
O1—C2	1.215 (2)	C6—H6A	0.9600
O2—C12	1.224 (2)	C6—H6B	0.9600
N1—C2	1.362 (2)	C6—H6C	0.9600
N1—C3	1.482 (2)	C7—H7A	0.9700
N1—C8	1.499 (2)	C7—H7B	0.9700
N2—C12	1.348 (2)	C8—C9	1.526 (2)
N2—C7	1.452 (2)	C8—C10	1.544 (2)
N2—C8	1.462 (2)	C9—H9A	0.9600
C1—C2	1.537 (2)	C9—H9B	0.9600
C1—H1	0.9800	C9—H9C	0.9600
C3—C4	1.527 (2)	C10—C11	1.528 (3)
C3—H3A	0.9700	C10—H10A	0.9700
C3—H3B	0.9700	C10—H10B	0.9700
C4—C7	1.522 (2)	C11—C12	1.499 (3)
C4—C6	1.524 (2)	C11—H11A	0.9700
C4—C5	1.528 (2)	C11—H11B	0.9700
C5—H5A	0.9600		
			
C2—N1—C3	121.54 (13)	C4—C6—H6C	109.5
C2—N1—C8	115.95 (13)	H6A—C6—H6C	109.5
C3—N1—C8	116.53 (12)	H6B—C6—H6C	109.5
C12—N2—C7	123.66 (15)	N2—C7—C4	108.86 (13)
C12—N2—C8	114.61 (15)	N2—C7—H7A	109.9
C7—N2—C8	121.72 (14)	C4—C7—H7A	109.9
C2—C1—Cl1	109.38 (12)	N2—C7—H7B	109.9
C2—C1—Cl2	107.50 (13)	C4—C7—H7B	109.9
Cl1—C1—Cl2	110.34 (9)	H7A—C7—H7B	108.3
C2—C1—H1	109.9	N2—C8—N1	107.82 (13)
Cl1—C1—H1	109.9	N2—C8—C9	110.68 (14)
Cl2—C1—H1	109.9	N1—C8—C9	110.48 (14)
O1—C2—N1	124.36 (16)	N2—C8—C10	101.88 (14)
O1—C2—C1	119.10 (15)	N1—C8—C10	112.40 (13)
N1—C2—C1	116.54 (14)	C9—C8—C10	113.16 (15)
N1—C3—C4	113.10 (13)	C8—C9—H9A	109.5
N1—C3—H3A	109.0	C8—C9—H9B	109.5
C4—C3—H3A	109.0	H9A—C9—H9B	109.5
N1—C3—H3B	109.0	C8—C9—H9C	109.5
C4—C3—H3B	109.0	H9A—C9—H9C	109.5
H3A—C3—H3B	107.8	H9B—C9—H9C	109.5
C7—C4—C6	110.55 (14)	C11—C10—C8	104.57 (15)
C7—C4—C3	107.05 (14)	C11—C10—H10A	110.8
C6—C4—C3	110.96 (14)	C8—C10—H10A	110.8
C7—C4—C5	109.74 (15)	C11—C10—H10B	110.8
C6—C4—C5	110.41 (16)	C8—C10—H10B	110.8
C3—C4—C5	108.05 (15)	H10A—C10—H10B	108.9
C4—C5—H5A	109.5	C12—C11—C10	104.01 (15)
C4—C5—H5B	109.5	C12—C11—H11A	111.0
H5A—C5—H5B	109.5	C10—C11—H11A	111.0
C4—C5—H5C	109.5	C12—C11—H11B	111.0
H5A—C5—H5C	109.5	C10—C11—H11B	111.0
H5B—C5—H5C	109.5	H11A—C11—H11B	109.0
C4—C6—H6A	109.5	O2—C12—N2	124.68 (19)
C4—C6—H6B	109.5	O2—C12—C11	126.95 (18)
H6A—C6—H6B	109.5	N2—C12—C11	108.34 (16)

### Hydrogen-bond geometry (Å, °)

**Table:**

*D*—H···*A*	*D*—H	H···*A*	*D*···*A*	*D*—H···*A*
C1—H1···O2^i^	0.98	2.23	3.200 (3)	170
C3—H3B···O2^i^	0.97	2.50	3.390 (2)	153

Symmetry codes: (i) −*x*, *y*+1/2, −*z*+1/2.
